# Response of Mechanical Properties in PVC-P GMB to Tensile Rate and Low Temperature

**DOI:** 10.3390/polym18151908

**Published:** 2026-08-03

**Authors:** Xinyan Li, Zhenxue Zhu, Jian Sun, Xianlei Zhang

**Affiliations:** 1 School of Water Conservancy, North China University of Water Resources and Electric Power, Zhengzhou 450046, China; lixinyan_0101@163.com; 2 Jilin Provincial Center for Flood and Drought Disaster Prevention, Changchun 130031, China; zhenxuered@163.com; 3 Jilin Province Water Resource and Hydropower Consultative Company, Changchun 130021, China; sj0408@163.com

**Keywords:** PVC-P geomembrane, low temperature, tensile rate, fracture strength, fracture strain, elastic modulus

## Abstract

Plasticized polyvinyl chloride (PVC-P) geomembranes (GMBs) are widely used in cold regions as impervious barriers, facing combined low-temperature and variable loading effects. However, design specifications rely on room-temperature tests at fixed rates, not reflecting actual conditions. This study systematically investigates the axial tensile properties of a 1.5 mm thick PVC-P GMB across eight temperatures (−40 °C to 20 °C) and five tensile rates (1–100 mm/min). A total of 211 uniaxial tensile tests were conducted using a low-temperature system with a servo-hydraulic machine and DIC extensometer. Nominal and true stress–strain curves were analyzed. Results show that fracture strength, fracture strain, and elastic modulus are highly sensitive to temperature and tensile rate, with a pronounced coupling effect between these two factors. Lower temperatures increase fracture strength and elastic modulus but reduce fracture strain, leading to brittle transition at −40 °C, especially at high rates. The fracture strength, fracture strain, and elastic modulus all increased with tensile rates at low tensile rates (1–20 mm/min). However, negligible difference in these parameters at high rates (20–100 mm/min) was observed. Elastic modulus follows a Boltzmann function with temperature, and fracture strain linearly correlates with temperature (R^2^ > 0.93). The mechanical properties measured at room temperature overestimate the deformability at low-temperature and hence underestimate the brittle failure risk. Therefore, future cold-region testing should adopt tensile rates of 10 or 20 mm/min, and temperature-rate coupled constitutive models should be developed. These findings provide essential data and guidance for material selection, design, and standard revision for PVC-P GMBs in cold-region applications.

## 1. Introduction

Plasticized polyvinyl chloride (PVC-P) geomembranes (GMBs) have been widely employed as critical impermeable barriers in hydraulic engineering, mining environmental protection, agricultural irrigation, and pumped storage hydropower projects in cold regions, owing to their excellent impermeability, high deformability, resistance to acid and alkali corrosion, construction convenience, and cost-effectiveness [1,2,3,4,5]. PVC-P GMBs have been successfully applied in several landmark infrastructure projects in China. For instance, the Jiangpinghe high concrete face rockfill dam in Hubei Province, with a water head exceeding 200 m, extensively utilizes a 2.5 mm thick PVC-P GMB as the primary impervious layer on the dam surface, relying on the material’s large deformation capacity to accommodate differential settlements. Located in high-altitude cold zones experiencing winter temperatures lower than −35 °C, Xinjiang’s Hami and Qinghai Golmud’s Nanshankou pumped storage power stations fully line their reservoir basins with PVC-P GMBs, creating flexible impervious systems resistant to frost heave and cyclic foundation deformations. In mining environmental protection, high-cold tailings ponds—such as the Pogo gold mine in Alaska and non-ferrous metal tailings ponds in northeastern China—utilize PVC-P GMBs to prevent toxic tailings wastewater from contaminating soil and groundwater. In high-cold hydropower projects, the Taishir RCC dam in Mongolia, located in an extreme low-temperature zone with a historical minimum of −51 °C, features a PVC-P GMB fully laid on the upstream face as the primary impervious structure, pioneering the application of full membrane impervious systems in high-cold RCC dams [6,7,8,9].

In the high-cold regions of northeastern, northwestern and the Qinghai–Tibet Plateau in China, where extreme winter temperatures commonly reach −30 to −40 °C, PVC-P GMBs are subjected to long-term freeze–thaw cycles while simultaneously enduring low-rate creep or instantaneous tensile loads caused by dam settlement, frost heave, and water fluctuations. The coupled effects of service temperature and loading rate can significantly alter the tensile mechanical properties of the GMB, inducing strain hardening and brittle cracking, and subsequent damage to the impervious layer—a key trigger for frequent failures in high-cold impervious projects.

Temperature is one of the most important environmental factors affecting the mechanical performance of GMBs [9,10,11]. Current national standards and ASTM testing protocols [12,13,14,15,16,17] predominantly measure the tensile properties of GMBs at room temperature (23 ± 2 °C) under fixed tensile rates, creating a substantial discrepancy between laboratory conditions and actual service environments in high-cold engineering applications. For polymeric GMBs, decreasing temperature generally increases stiffness and strength while reducing ductility and fracture strain [18]. For PVC-P GMBs in particular, low temperatures result in increased tensile modulus and reduced elongation, whereas elevated temperatures produce the opposite effect [4]. As a typical viscoelastic material, PVC-P exhibits molecular chain segment motion that is highly dependent on both temperature and loading duration. Low temperatures constrain chain segment mobility, while the loading rate alters the relaxation time of molecular chains. Their interaction causes significant variations in fracture strength, fracture strain, and elastic modulus.

The influence of loading rate on GMB mechanical behavior has been extensively documented. Merry and Bray [19] demonstrated that the time-dependent mechanical response of HDPE GMBs is highly sensitive to strain rate, with both initial modulus and maximum stress decreasing significantly as strain rate decreases. Akel et al. [18] demonstrated that the tensile properties of PVC-P GMBs across a wide range of displacement rates (0.01 to 500 mm/min), revealing that tensile modulus, strength, and fracture strain all increase with increasing loading rate, with the material exhibiting a 214% increase in true fracture strength as the rate rises from 0.01 to 500 mm/min. Lamri et al. [20] confirmed for HDPE GMBs that strain rate and temperature have coupled effects on mechanical behavior, with viscosity significantly reduced at elevated temperatures and the material displaying a stiffer response at increased strain rates. Tahir et al. [6] systematically investigated the tensile behavior of seven types of GMBs (HDPE, EPDM, PVC, FPO, bituminous, and reinforced PVC) across a wide speed range from 0.01 to 500 mm/min, finding that internal temperature rises due to self-heating can reach up to 37 K at high loading rates (500 mm/min), with self-heating and viscous effects jointly dominating the high-rate tensile response. They further demonstrated that for test speeds of 10 mm/min or lower, the internal temperature change remains insignificant (<2 K), suggesting that lower-rate tests better reflect intrinsic material behavior without thermal interference [6].

Regarding low-temperature performance, Somuah et al. [21] conducted impact and tensile tests on polyethylene GMBs and found that the ductile-to-brittle transition temperature (DBTT) occurred between −16 °C and −27 °C, significantly above the glass transition temperature (Tg) of polyethylene (which typically ranges from −60 to −115 °C). This finding underscores that Tg alone is not a reliable predictor of cold-weather fracture behavior, and that other factors—such as loading rate, stress concentration, and surface defects—play critical roles in cold-temperature failures. Zhang et al. [22] conducted axial tensile tests on PVC-P GMBs over a temperature range of −40 to 60 °C, revealing that low temperatures reduce elongation at break and weaken flexibility, while the elastic modulus increases significantly with decreasing temperature, and established a Boltzmann function to describe the temperature-dependent evolution of Young’s modulus. Studies on PVC-P GMBs installed on Italian dams over a 25-year period have also shown that temperature directly affects mechanical properties: high temperatures result in reduced tensile modulus and increased elongation, while low temperatures produce the opposite effect [4]. Furthermore, the durability of PVC-P GMBs in cold environments is closely related to plasticizer retention, as plasticizer loss increases rigidity and reduces low-temperature flexibility [16,23].

Despite these advancements, existing research has predominantly focused on mechanical testing of PVC-P GMBs at room temperature or single temperature conditions. Axial tensile tests under wide temperature range (from −40 °C to room temperature) and multiple tensile rates remain scarce. The transition characteristics from ductile to brittle and the evolution of mechanical properties with tensile rate in the low-temperature region are rarely quantified. For projects in high-cold regions, mechanical parameters measured at room temperature are still adopted in design, which overestimate the plastic reserve and underestimate the risk of brittle fracture at low temperatures, posing potential dangers to impervious safety.

This study selects a commercial 1.5 mm thick PVC-P GMB and employs a low-temperature specialized testing platform integrating a high and low temperature test chamber, a 30 kN servo-hydraulic tensile testing machine, and a digital image correlation (DIC) video extensometer. Comprehensive tests were conducted across eight temperature gradients ranging from −40 °C to 20 °C and five tensile rates from 1 to 100 mm/min, yielding a total of 211 valid datasets. These results fill the systematic data gap concerning the axial tensile properties of PVC-P under various temperature gradients and tensile rates. Both engineering stress–strain and true stress–strain are analyzed to investigate the effects of temperature, tensile rate, and their coupled effects on fracture strength, fracture strain, and elastic modulus, and clarify the ductile-to-brittle transition range of PVC-P GMBs at low temperatures. The study quantitatively revealed that, at the temperatures below the glass transition temperature, both fracture strength and fracture strain exhibit an anomalous decrease with increasing tensile rate, a phenomenon for which a physical explanation is provided. Furthermore, two empirical models were established: a linear relationship between fracture strain and temperature (R^2^ > 0.934), and a Boltzmann function for elastic modulus as a function of temperature (R^2^ > 0.994). These findings will provide insight into the mechanical behavior of PVC-P GMBs under high-cold conditions, providing experimental evidence and support for material selection, structural optimization, and revision of testing standards for GMBs in reservoirs, irrigation projects, and tailings ponds in cold regions.

## 2. Materials and Methods

### 2.1. Materials

**Figure 1:**
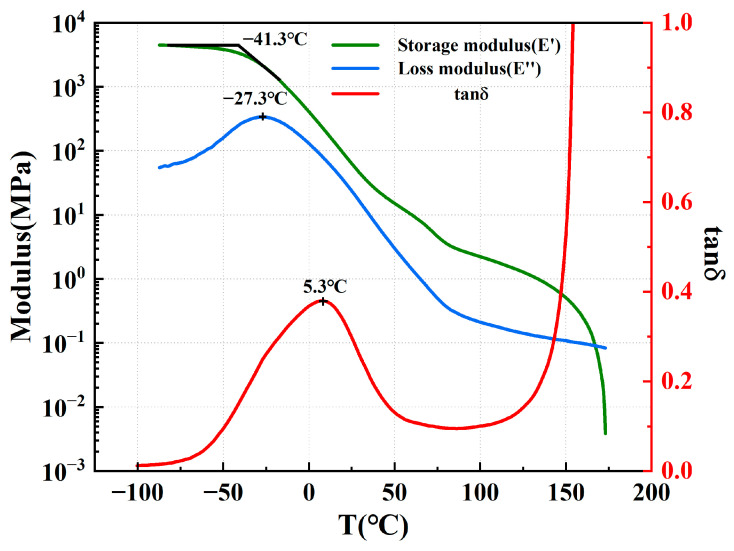
Temperature dependence of storage modulus (E′), loss modulus (E″) and loss factor (tan δ) of PVC-P GMB.

The test material was supplied by Wuxi Xingwei Geosynthetics Co., Ltd., Wuxi, China. The PVC-P GMB used was produced via a calendering process. Its main constituents, by mass percentage, are 57.4% PVC resin, 20.0% plasticizer, 0.5% antioxidant, 2.1% stabilizer, and 20.0% inorganic filler. The material density is 1.47 g/cm^3^. Tg determined by Dynamic Mechanical Analysis (DMA) measurements is approximately −27.3 °C, as shown in Figure 1. Each roll measures 50.0 m in length and 2.0 m in width. The key physical and mechanical properties are summarized in Table 1.

**Figure 2:**
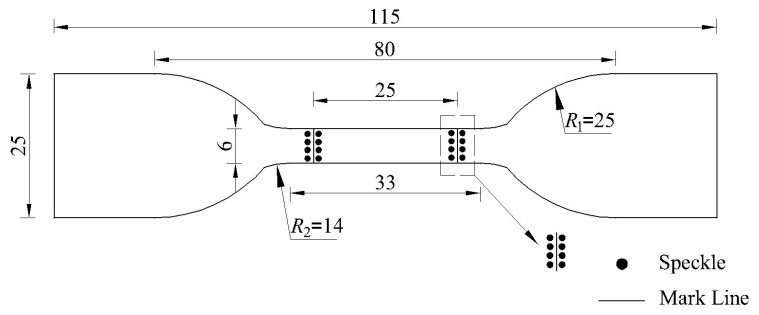
Schematic diagram of the dumbbell-shaped specimen (unit: mm).

The dumbbell-shaped specimen measured 115 mm in length and 25 mm in width. The distance between the two marking points on the narrowest section is 25 mm. The calibration segment is covered with a white permanent marker, while the rest is marked with a black permanent marker, the mark line is determined, then the speckles are drawn symmetrically on either side of the line, as shown in Figure 2.

### 2.2. Testing System for the Low-Temperature Axial Tensile Mechanical Properties of GMBs

**Figure 3:**
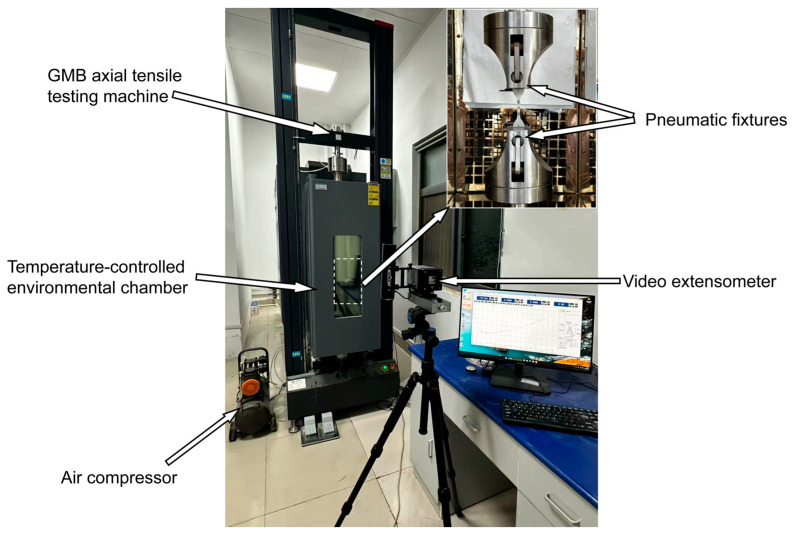
Testing system for the low-temperature axial tensile mechanical properties of GMBs.

The test system (Kaier Measurement and Control Technology Co., Ltd., Tianjin, China) for assessing the low-temperature axial tensile mechanical properties of GMB is composed of a GMB axial tensile testing machine, a temperature-controlled environmental chamber and a video extensometer, as illustrated in Figure 3.

#### 2.2.1. GMB Axial Tensile Testing Machine

The axial tensile testing machine for GMB has a maximum tensile force of 30 kN, with a measurement error controlled to within ±0.5%; the deformation measurement system has a measurement error of ±0.5%; the displacement control rate ranges from 0.001–1000 mm/min; the rate control accuracy is within ±0.5% of the set value when the rate is <0.5 mm/min, and within ±0.2% of the set value when the rate is ≥0.5 mm/min; the displacement reading error is within ±0.5%, and the displacement resolution is 0.015 μm. The main unit features an effective tensile stroke of 2000 mm, a width of 550 mm, overall dimensions of 950 mm × 550 mm × 2690 mm, operates on AC 220 V ± 10% at 50 Hz with a power of 750 W, weighs 350 kg, employs a bottom-space testing configuration, and is equipped with a 30 kN load sensor, an AC servo motor, and a servo controller to ensure stable and precise loading during tests.

The GMB axial tensile testing machine utilizes high- and low-temperature pneumatic fixtures, serving as a critical component for low-temperature testing. Powered by compressed air, the pneumatic system utilizes pneumatic control combined with a mechanical force-amplification mechanism to achieve rapid, uniform, and non-destructive clamping of PVC-P GMB specimens while maintaining constant clamping force throughout the tensile test. This prevents specimen slippage at the fixture contact point due to reduced thickness at the application site, thereby ensuring accurate test results. The pneumatic fixture is controlled by an air compressor capable of delivering a stable pressure range of 0.4–0.6 MPa.

#### 2.2.2. Temperature-Controlled Environmental Chamber

The temperature control system employs a high-low temperature chamber with a fully integrated vertical design that completely encloses the specimen within a temperature-controlled cavity. The internal temperature ranged between −40 °C and 350 °C, with a precision of ± 0.1 °C. The 304 stainless steel chamber measures 400 mm × 330 mm × 1200 mm (length × width × height), featuring forced air circulation via a centrifugal fan. A hollow insulated tempered glass panel measuring 200 mm × 580 mm is installed at the lower center of the door. The temperature control system utilizes a Proportional-Integral-Derivative (PID) closed-loop control scheme, supporting constant temperature, programmed heating and cooling, and high-low temperature cycling. It ensures outstanding temperature uniformity and negligible temperature fluctuation, allowing accurate simulation of diverse service temperature conditions, including extreme low, ambient, and high temperatures.

#### 2.2.3. Video Extensometer

The experiment employed a non-contact video extensometer (LVE-MAX 5M) to measure the displacement of GMBs during tensile testing. Based on DIC technology, this device tracks the displacement of feature points on the specimen, enabling non-contact, high-precision, and full-field strain measurement while effectively overcoming the limitations of traditional contact extensometers, such as slippage, specimen damage, and poor adaptability to large deformations.

The hardware comprises an industrial camera (resolution: 2448 × 2048 pixels; pixel size: 4.8 μm) paired with a 25 mm lens and a blue LED light source with a central wavelength of 450 nm. At a working distance of 300 mm, the field of view is approximately 141 mm × 117 mm, within which the displacement measurement accuracy reaches ±1.5 μm and the maximum resolution is 0.8 μm under standard viewing conditions. The strain measurement range spans from 0.5% to 1000%, satisfying the Class 0.5 accuracy in ISO 9513 standard [26]. During software processing, a subset size of 21 × 21 pixels, a step size of 5 pixels, and frames per second of 29 FPS are employed to acquire displacement and strain throughout the tensile testing of the GMBs.

### 2.3. Methods

To investigate the effects of tensile rate and low temperature on the mechanical properties of PVC-P GMBs, five tensile rates (1, 10, 20, 50, and 100 mm/min) and eight temperatures (−40, −30, −20, −10, 0, 4, 10, and 20 °C) were adopted. For each condition, five parallel tensile tests were performed. The raw data were screened using the following outlier exclusion criteria: data points exhibiting deviations of more than ±2 standard deviations from the mean of the five parallel tests were identified as outliers and removed. Subsequently, one to two additional parallel tests were conducted to compensate for the excluded data and ensure a sufficient sample size, ultimately resulting in 211 valid tests in total. The data for each condition was averaged as the mechanical property.

The testing protocol consisted of the following steps:

1. Specimen preparation: The dumbbell-shaped specimens were cut from the PVC-P GMB along the machine direction using an electro-hydraulic cutting press with a matching die. The gauge length was marked and the actual thickness was measured with a digital thickness gauge. Speckle patterns were then uniformly applied to the effective test area, ensuring moderate size, even distribution, and clear edges of speckles.
2. Temperature conditioning: Prior to testing, the marked specimens were placed in a temperature-controlled environmental chamber at the test temperature for no less than 48 h to ensure thermal equilibrium throughout the specimens.
3. Specimen mounting: After the internal temperature stabilized at the preset temperature, the chamber door was opened. The prepared specimen was mounted centrally and clamped firmly in the pneumatic grips to align its axis with tension and avoid eccentricity or slippage. The door was then locked to prevent air convection from affecting the temperature during subsequent testing.
4. Loading and data acquisition: The video extensometer was synchronized with the axial tensile testing machine for data acquisition. Following tensile rate setup, the axial tension was applied until specimen fracture. The system synchronously recorded test time, loads, and deformation in the gauge length throughout the tensile process.

All parallel specimens under each condition followed the identical procedure (specimen preparation, temperature conditioning, specimen mounting, loading and data acquisition) to ensure consistent operating environments and effectively reduce systematic measurement errors.

## 3. Results and Analysis

### 3.1. Test Results and Preliminary Analysis

The low-temperature testing system recorded the applied force and the displacement throughout the axial tensile tests, yielding the force–displacement curve within the gauge length. The nominal stress–strain relationship was then derived using Equations (1) and (2). Nominal stress is calculated based on the constant initial cross-sectional area, without considering the reduction in cross-sectional area during tensile deformation. Such curves are widely employed in engineering practice, owing to its simple calculations.

(1) $$\varepsilon_{aE}=\frac{\Delta L}{L_{0}}=\frac{L_{f}-L_{0}}{L_{0}}\times 100$$

where $\varepsilon_{aE}$ is the nominal axial strain (%), $L_{0}$ is the initial gauge length (mm), and $L_{f}$ is the gauge length after deformation (mm).

(2) $$\sigma_{E}=\frac{F_{t}}{A_{0}}$$

where $\sigma_{E}$ is the nominal stress (MPa), $F_{t}$ is the tensile force at time t + Δt (kN), and $A_{0}$ is the initial cross-sectional area in the gauge length (mm^2^).

**Figure 4:**
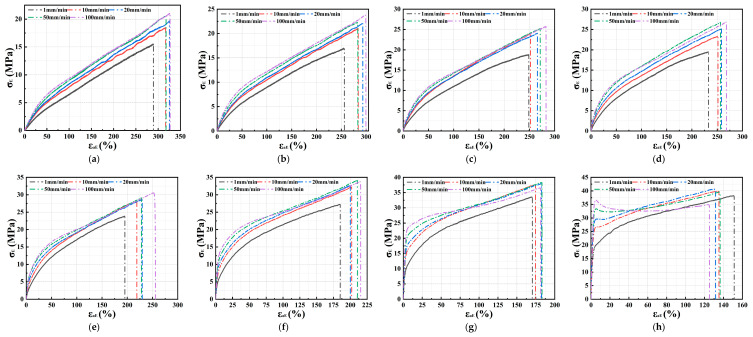
Nominal stress–strain curves at different tensile rates: (**a**) 20 °C; (**b**) 10 °C; (**c**) 4 °C; (**d**) 0 °C; (**e**) −10 °C; (**f**) −20 °C; (**g**) −30 °C; (**h**) −40 °C.

Figure 4 presents the nominal stress–strain curves for the tested PVC-P GMBs at all test temperatures, under tensile rates of 1, 10, 20, 50, and 100 mm/min. All curves exhibit an initial quasi-linear elastic stage followed by a strain-hardening plastic stage until tensile rupture, showing a rapid increase in stress and a steep slope in the low-strain stage and diminished increase rate in the high-strain stage. At all test temperatures, the tensile strength of the GMBs show significant dependence on tensile rate, manifested as an increase in nominal stress with increasing tensile rate at the same strain level. Both the fracture strength and fracture strain increase monotonically with rising tensile rate. This can be attributed to insufficient molecular chain relaxation under rapid loading. The curves at high tensile rates (50 and 100 mm/min) exhibited similar shapes with close nominal fracture strains and strengths. The curves at medium rates (10 and 20 mm/min) shared comparable patterns but with noticeable fluctuations. By comparison, the curve at the low rate (1 mm/min) was markedly lower across the full strain range, with smaller nominal fracture strain and strengths. At 20 °C (Figure 4a), as the tensile rate increased from 1 mm/min to 10, 20, 50, and 100 mm/min, the fracture strength of the PVC-P GMB rose from 15.34 MPa to 18.39, 19.69, 20.52, and 21.07 MPa, respectively, while the nominal strain increased from 289.36% to 316.79%, 325.17%, 317.65%, and 326.79%.

Table 2 and Table 3 present the nominal fracture strains and strengths of the PVC-P GMBs at different tensile rates. The tensile rate of 50 mm/min, specified as the standard testing speed for uniaxial tensile tests on geomembranes by ASTM standards (ASTM D638 [17]), is adopted as the reference condition to calculate the deviations at other rates. ε_1−50_, ε_10−50_, ε_20−50_, and ε_100−50_ represent the differences in fracture strain between the test results obtained at tensile rates of 1, 10, 20, and 100 mm/min and the values at 50 mm/min. Similarly, σ_1−50_, σ_10−50_, σ_20−50_ and σ_100−50_ denote the differences in fracture strength between the measurements at 1, 10, 20, and 100 mm/min and the corresponding values at 50 mm/min.

At 10 °C (Figure 4b), the differences in fracture strain and fracture strength between the high-rate range (50 and 100 mm/min) were 16.3% and 1.48 MPa, respectively—a marked increase compared with the values of 9.14% and 0.55 MPa observed at 20 °C. Both fracture strain and fracture strength at 50 mm/min better align with those measured under medium tensile rates. At 4 °C (Figure 4c), the nominal stress–strain curves in the medium and high rates (10–100 mm/min) exhibited greater dispersion. The nominal fracture strain and strength at 20 mm/min and 50 mm/min were relatively close, with differences of 10.06% and −0.27 MPa, respectively. At 0 °C (Figure 4d), the nominal stress–strain curves followed the same three-range pattern observed at 20 °C. At −10 °C (Figure 4e), the nominal fracture strain and strength at 20 mm/min and 50 mm/min remained comparable, with differences of 1.41% and −0.78 MPa, respectively. At −20 °C (Figure 4f), the curves again exhibited the three-range pattern observed at 20 °C. However, the fracture strength at 50 mm/min exceeded that at 100 mm/min by 4.85 MPa, while the fracture strain continued to increase with increasing tensile rate. At −30 °C (Figure 4g), the fracture strengths reached 37.04, 37.51, and 38.18 MPa at 10, 20, and 50 mm/min, respectively, all exceeding the 36.38 MPa at 100 mm/min. The curves at different tensile rates differed considerably in the early stage (nominal strain < 75%), with a distinct yield stage at 100 mm/min; in the subsequent large-deformation stage, the nominal stress–strain curves at 10, 20, and 50 mm/min largely coincided, and the increase in nominal stress was greater than that at 100 mm/min. At −40 °C (Figure 4h), the nominal stress–strain curves at the medium-to-high tensile rates changed, with a pronounced yield stage: the nominal stress rose rapidly in the early stage, then decreased, before rising gradually again until specimen fracture. In contrast to the rate effect observed at other temperatures, the fracture strain at 1 mm/min increased markedly compared with that at medium and high rates, reaching 151.31%, whereas the nominal fracture strain at 100 mm/min was the lowest, at only 125.14%. The nominal fracture strains and strengths at 10, 20, and 50 mm/min were relatively close to one another.

**Figure 5:**
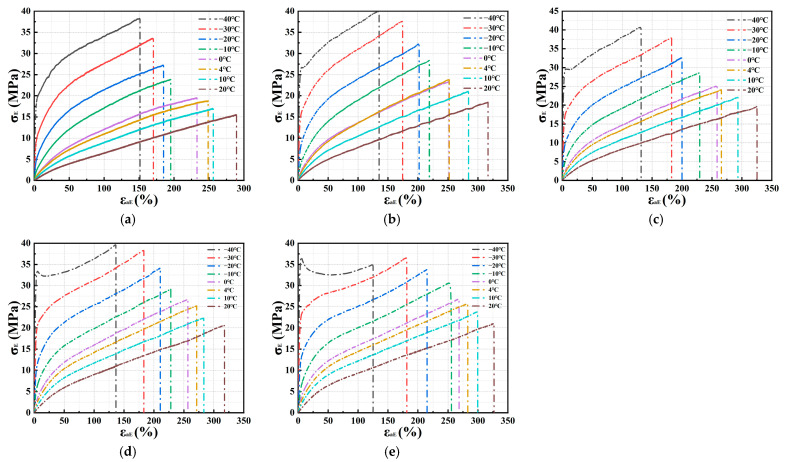
Nominal stress–strain curves at different test temperatures: (**a**) 1 mm/min; (**b**) 10 mm/min; (**c**) 20 mm/min; (**d**) 50 mm/min; (**e**) 100 mm/min.

Figure 5 presents the nominal stress–strain curves of the PVC-P GMBs across the temperature range of −40 °C to 20 °C, clearly illustrating the effect of temperature on the mechanical properties. This pattern is highly consistent with the temperature-dependent viscoelastic behavior of polymeric materials. Under all test conditions, the fracture strength of the PVC-P GMBs decreased significantly with increasing temperature, while its fracture strain increased substantially with rising temperature. This trend clearly reveals the typical temperature-softening effect, characterizing the mechanical properties of polymeric materials under thermally activation and their sensitivity to temperature. Taking a tensile rate of 1 mm/min (Figure 5a) as an example, the fracture strength at −40 °C reached 37.06 MPa, whereas at 20 °C it dropped to 15.34 MPa—a decrease of over 58%— while the fracture strain increased from 151.31% to 289.36%, representing an increase of more than 91%. These observations demonstrate that temperature exerts a significant influence on both fracture strength and strain: from low temperatures to room temperature, the material’s strength decreases substantially while its ductility improves markedly. At a high tensile rate of 100 mm/min (Figure 4e), the fracture strain at −40 °C was only 125.14%, whereas at 20 °C it surged to over 300%, confirming the enhancement of elevated temperature on plastic deformation.

The morphology of the nominal stress–strain curves at −40 °C differs markedly from that at other temperatures, with this distinction being particularly pronounced at tensile rates of 10, 20, 50, and 100 mm/min. In the early stage of tensile loading, the curve rises rapidly in an approximately linear manner; the stress growth rate during the low-strain phase is substantially higher than that of the curves at other temperatures, with no gradual transition zone. The PVC-P GMBs exhibit exceptionally high rigidity: after reaching its peak, the nominal stress declines before gradually increasing again until fracture. The broad plastic deformation plateau characteristic of the curves at higher temperatures is absent, and the proportion of the elastic deformation region is significantly increased.

As temperature decreases, the thermal mobility of polymer chains gradually diminishes, and the internal structure of the GMB transitions from a highly elastic state to a glassy state [22]. This leads to reduced molecular chain flexibility, increased fracture strength, and markedly diminished plastic deformation, exhibiting clear hardening and embrittlement. This transition constitutes the fundamental mechanism underlying the temperature dependence of polymeric materials and directly affects their engineering performance. At the relatively higher temperatures of 10 °C and 20 °C, the molecular segments of the PVC-P GMBs acquire sufficient thermal energy, enhancing their mobility; this reduces fracture strength and significantly improves ductility. Consequently, the fracture strain is considerably higher than that under low temperatures, exhibiting typical viscoelastic behavior characterized by viscous flow and elastic recovery. This indicates that using room temperature (20 °C) to evaluate the mechanical properties of PVC-P GMBs intended for low-temperature service has significant limitations.

### 3.2. Analysis and Discussion

#### 3.2.1. True Stress and True Strain

While the nominal stress–strain curves provide fundamental and crucial data for the experiments, the axial true stress–strain curves [2] offer deeper insights into the mechanical properties of the PVC-P GMBs, enabling a thorough analysis of the true fracture strength and strain. Notably, the axial true stress accounts for the changes in the cross-sectional area of the specimen during tensile deformation. The true stress–strain curves in this study was derived under the constant-volume assumption, which is applicable to the large-deformation tensile behavior of PVC-P GMBs. PVC-P is a highly elastic viscoplastic polymer; its high plasticizer content renders it approximately incompressible, with a Poisson’s ratio ν ≈ 0.5 during tensile loading and a volume change rate of <0.8%. Consequently, computational errors arising from volume contraction are negligible [19]. Wesseloo et al. [27] adopted this same assumption when developing a strain-rate-dependent constitutive model for HDPE GMBs, confirming that uniaxial tensile testing of both PVC and HDPE GMBs can be treated as constant-volume processes. This assumption is also uniformly employed in existing studies on the tensile behavior of GMBs and flexible polymers. Compared with results obtained using a Poisson’s ratio segmentation correction, the relative error of the constant-volume method is <1.2%, which meets the precision requirements for engineering applications. Therefore, this simplified approach is adopted herein to derive the true stress and strain formulas. The true strain is given by Equation (3):

(3) $$\varepsilon_{aT}=\int_{L_{0}}^{L_{t}}\frac{dL}{L}=\ln\left(\frac{L_{t}}{L_{0}}\right)=\ln\left(\frac{L_{0}+\Delta L}{L_{0}}\right)=\ln\left(1+\varepsilon_{aE}\right)$$

where $L_{0}$ is the initial gauge length (mm), $L_{t}$ is the gauge length after deformation at time t + Δt (mm), and L is the gauge length at a given instant during tensile loading (mm).

Assuming constant volume within the gauge length throughout the axial tensile deformation process, and denoting the initial cross-sectional area as $A_{0}$, the initial length as $L_{0}$. At any instant time (t) during axial tensile deformation, the cross-sectional area is $A_{t}$ and the deformed gauge length is $L_{t}$, we obtain:

(4) $$A_{t}=A_{0}L_{0}/L_{t}$$

The true stress at time (t) can be expressed as:

(5) $$\sigma_{tT}=\frac{F_{t}}{A_{t}}=\frac{F_{t}L_{t}}{A_{0}L_{0}}=\frac{F_{t}(L_{0}+\Delta L)}{A_{0}L_{0}}=\frac{F_{t}}{A_{0}}\frac{L_{0}+\Delta L}{L_{0}}=\frac{F_{t}}{A_{0}}\left(1+\varepsilon_{aE}\right)=\sigma_{E}\left(1+\varepsilon_{aE}\right)$$

where $F_{t}$ is the tensile force at time t + Δt (kN).

The nominal stress–strain offers significant advantages in engineering practice due to its computational simplicity and direct compatibility with current design codes. However, it suffers from issues such as stress data distortion and overestimated deformation, facing challenges in constructing accurate constitutive models. In contrast, the true stress–strain curve considers the cross-sectional area changes during tensile testing, enabling a more accurate mechanical response under large deformation. It therefore serves as a critical basis for finite element simulations and constitutive parameter inversion, but requires higher precision in experimental data acquisition and processing. The fundamental difference between the two curves lies in their calculation bases: the former emphasizes convenience and standardization for engineering applications, whereas the latter aligns more closely with the physical reality of the deformation process.

Previous studies have often employed Poisson’s ratio-based methods to derive true stress–strain relationships under various tensile conditions; however, this approach is applicable only to the linear elastic stage and thus has considerable limitations. The constant-volume method adopted in this study overcomes the shortcomings of the Poisson’s ratio approach and enhances the reliability of the results. Based on Equations (3) and (5), the true stress–strain curves throughout the tensile process were derived.

#### 3.2.2. Effect of Tensile Rate on the Mechanical Properties of GMBs

**Figure 6:**
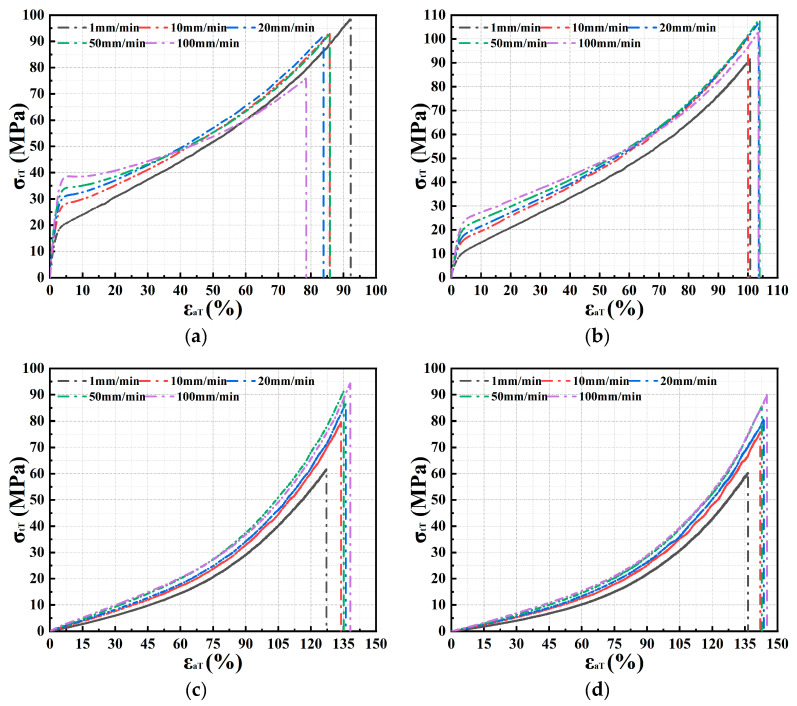
True stress–strain curves at different tensile rates: (**a**) −40 °C; (**b**) −30 °C; (**c**) 10 °C; (**d**) 20 °C.

Figure 6 presents the true stress–strain curves for the PVC-P GMBs at all tensile rates tested, differing markedly from the nominal stress–strain curves in Figure 4. In the late deformation stage, the slope of the true stress–strain curve increases progressively, whereas that of the nominal stress–strain curve remains relatively unchanged—this is because the nominal stress is calculated based on the constant cross-sectional area, neglecting the reduction in width or thickness during deformation. As tensile strain increases, the true stress rises at a considerably higher rate than the nominal stress. At a tensile rate of 1 mm/min, the nominal and true fracture strengths were 37.06 MPa/98.10 MPa at −40 °C (Figure 4h and Figure 6a), 33.07 MPa/91.48 MPa at −30 °C (Figure 4g and Figure 6b), and 15.34 MPa/60.10 MPa at 20 °C (Figure 4a and Figure 6d). True fracture strength was substantially higher than nominal fracture strength in both cases. Progressive cross-sectional contraction with rising tensile strain enlarges the difference between true and nominal stress fracture strength over the tensile test.

The fracture strength of the PVC-P GMBs increases with increasing tensile rate (Figure 6c,d). The curves at different tensile rates exhibit only minor differences in the early stage (strain < 100%); however, in the subsequent large-deformation stage, the stress show accelerated growth rates for the higher tensile rates, and the gap relative to the lower-rate curves widens rapidly, with the fracture strength under high rates significantly exceeding that under low-rate conditions. Taking the true stress–strain curve at 10 °C and 100 mm/min as an example: when the true strain increased from its initial value to 100%, the stress increased by 44.61 MPa; when the true strain further increased to the fracture true strain of 138.1%, the stress rose to 94.77 MPa, representing an increase of 49.77 MPa. In the late stage, despite only a limited increase in strain, the stress rises sharply until fracture. This behavior is attributed to the high tensile rate suppressing the relaxation and slippage of polymer chain segments; the molecular chains are unable to achieve full orientation and plastic deformation, which substantially enhances the material’s instantaneous load-bearing capacity, manifested as higher fracture strength and stress increase.

Table 4 lists the fracture strength of the PVC-P GMBs at all test temperatures and tensile rates. The fracture strength exhibits distinct characteristics with respect to tensile rate. In the low tensile rate (1–20 mm/min), the fracture strength increases rapidly and substantially with increasing tensile rate, contributing significantly to the rate-hardening effect. For example, at −30 °C, when the tensile rate increased from 1 mm/min to 20 mm/min, the fracture strength rose from 91.48 MPa to 106.63 MPa, representing an increase of 16.56%; at 20 °C, it increased from 60.10 MPa to 81.21 MPa, corresponding to a 35.12% increase. The GMB exhibits high sensitivity to tensile rates, with the increase rate of fracture strength markedly higher than that in the medium-to-high rates. This indicates that even a modest increase in tensile rate within the low rates can effectively activate the strain hardening of the PVC-P GMBs, thereby rapidly enhancing its ultimate load-bearing capacity.

At medium–high tensile rates (20–100 mm/min), the growth of fracture strength tends to saturate with drastically reduced rate sensitivity. Under certain test conditions, a minor drop in fracture strength is occasionally observed. At −20 °C, when the rate raised from 20 mm/min to 100 mm/min, the fracture strength increased from 97.72 MPa to 106.33 MPa, representing a change of less than 9%. At 10 °C, the fracture strength rose from 86.31 MPa to 94.38 MPa, an increase of merely 9.35%, which is far below the growth rate in the low-rate range. This behavior suggests that once the tensile rate exceeds a certain threshold, the rate-hardening effect gradually stabilizes, and further increases in tensile rate exert only a limited influence on enhancing the fracture strength.

Table 5 presents the fracture strains of the PVC-P GMBs at various tensile rates. In the low rates (1–20 mm/min), the fracture strain shows a pronounced increase as the tensile rate rises, which primarily contributes to plastic deformability. For example, at −20 °C, when the tensile rate increased from 1 mm/min to 20 mm/min, the fracture strain rose from 105.47% to 110.17%, representing an increase of 4.46%; at −10 °C, it increased from 109.30% to 118.20%, corresponding to an increase of about 8.14%; and at 10 °C, it increased from 127.11% to 136.05%, an increase of approximately 7.03%. In this regime, the GMB exhibits considerable sensitivity to changes in loading rate, indicating that an increase in tensile rate within the low-rate can effectively promote the mobility of molecular chain segments, thereby enhancing the material’s plastic deformation capacity.

In the medium-to-high-rate regime (20–100 mm/min), the increase in fracture strain generally plateaus, and rate sensitivity diminishes notably; under certain test conditions, only minor fluctuations are observed. For example, at 0 °C, the fracture strain increased from 127.41% to 129.83%, a rise of only 1.90%; at 10 °C, it increased from 136.05% to 138.08%, an increase of merely 1.50%; and at 20 °C, when the tensile rate was raised from 20 mm/min to 100 mm/min, the fracture strain increased only from 143.63% to 145.62%, representing an increment of less than 1.4%. This behavior suggests that once the tensile rate reaches a certain threshold, the material’s plastic deformation capacity approaches saturation, and further increases in the tensile rate exert only a limited influence on enhancing the fracture strain.

Elastic modulus, a key indicator for evaluating elastic deformation resistance, varies markedly with temperature and tensile rate. The elastic modulus was determined using the 0.2% offset method based on selected typical true stress–strain curves. A least-squares linear fit was performed on the initial linear deformation segment, starting from the origin; only the linear interval with a coefficient of determination R^2^ ≥ 0.995 was retained to ensure fitting accuracy. This fitted line was then translated along the positive direction of the true strain axis by 0.2%. The intersection point between the translated line and the original true stress–strain curve was defined as the yield characteristic point of the material. The ratio of true stress to true strain at this point was taken as the elastic modulus of the PVC-P GMB.

**Figure 7:**
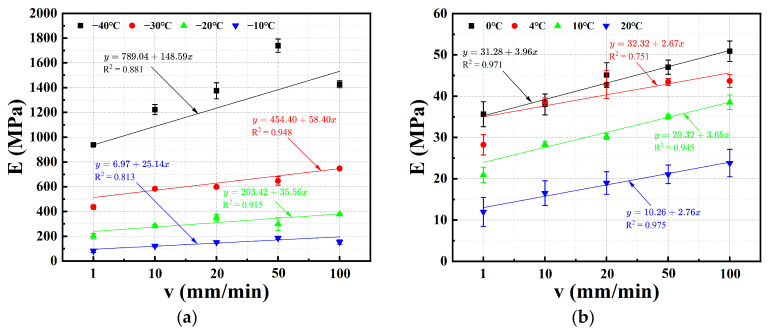
Distribution of elastic modulus and temperature at different tensile rates: (**a**) −40, −30, −20, −10 °C; (**b**) 0, 4, 10, 20 °C.

As shown in Figure 7, the elastic modulus generally exhibits an upward trend with increasing tensile rate. As the tensile rate rises from 1 mm/min to 100 mm/min, the modulus increases to varying degrees across all temperature levels, with this trend being particularly pronounced at lower temperatures. In low-temperature environments such as −40 °C and −30 °C, the elastic modulus shows a marked upward trend with increasing tensile rate, accompanied by a substantial rate of increase. In the temperature range of −20 °C to −10 °C, the influence of tensile rate on the elastic modulus persists but is considerably attenuated, with the modulus remaining within the 100–200 MPa range and the amplitude of variation diminishing as the rate changes. When the temperature rises to 0 °C and above (0 °C, 4 °C, 10 °C, 20 °C), the effect of tensile rate on the elastic modulus virtually disappears. Under these conditions, the modulus consistently remains at an extremely low level (approximately 0–50 MPa), and the data points exhibit virtually no discernible fluctuations as the tensile rate varies from 1 mm/min to 10 mm/min. This indicates that, within this temperature range, the mobility of molecular chain segments in the PVC-P GMBs are significantly enhanced, and the elastic modulus is no longer sensitive to tensile rate.

According to domestic and international standards for uniaxial tensile testing of GMBs, the commonly adopted tensile rate is 50 mm/min (ASTM D638 [17]), whereas laboratory tensile tests are typically conducted at a rate of 1 mm/min [19]. However, preliminary experimental results indicate that a rate of 1 mm/min is excessively low, prolonging test durations and introducing creep deformation [28]. Such creep behavior can distort the intrinsic mechanical properties of the PVC-P GMBs [25]. Conversely, a rate of 100 mm/min is overly high, not only exceeding the typical engineering loading rates but also causing unstable specimen fracture and increased data scatter, failing to reliably capture the material’s mechanical response. Therefore, in this study, tensile rates of 10 mm/min and 20 mm/min were selected as representative. These two rates fall within a reasonable range for both routine GMB testing and actual service conditions, thereby circumventing both the creep interference associated with extremely low rates and the instability issues inherent to high rates. Moreover, the moderate gradient between these two rates enables a clear discrimination of the evolution of fracture strength, fracture strain, and stress–strain response under different loading conditions.

#### 3.2.3. Effect of Temperature on the Mechanical Properties of GMBs

**Figure 8:**
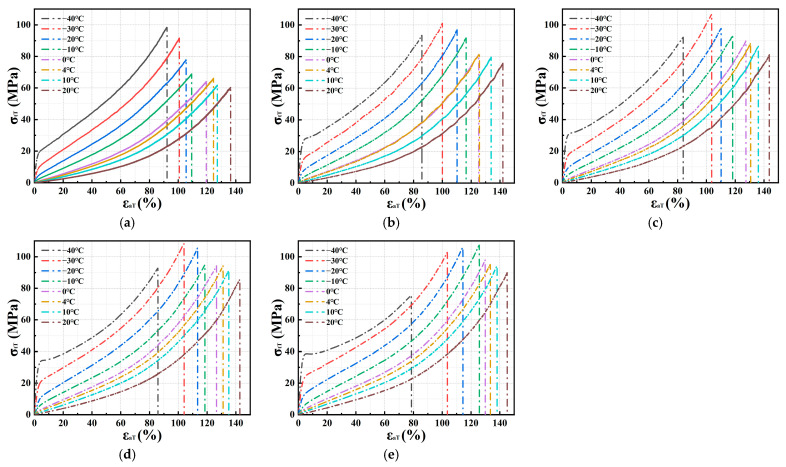
True stress–strain curves at different test temperatures: (**a**) 1 mm/min; (**b**) 10 mm/min; (**c**) 20 mm/min; (**d**) 50 mm/min; (**e**) 100 mm/min.

Figure 8 presents the stress–strain curves of the PVC-P GMBs at various temperatures. The test temperature exerts a significant effect on the mechanical behavior of the PVC-P GMBs, and this effect is clearly coupled with the tensile rate. At moderate and high tensile rates, the fracture strength first increases and then decreases as temperature declines. At the low tensile rate of 1 mm/min, fracture strength rises monotonically with decreasing temperature. Taking a tensile rate of 20 mm/min as an example (Figure 8c), the fracture strength is 81.21 MPa at 20 °C and increases to 106.63 MPa at −30 °C by 31.30%; at −40 °C, the fracture strength declines to 92.16 MPa, close to the value at −10 °C. At a tensile rate of 1 mm/min (Figure 8a), the fracture strength at 20 °C is 60.21 MPa, while at −40 °C it reaches 98.16 MPa, also corresponding to an increase of more than 63%, at −30 °C it reaches 91.48 MPa, also corresponding to an increase of more than 52%. This anomalous behavior originates from the substantial suppression of molecular chain mobility at ultra-low temperatures. The test temperature of −40 °C is considerably lower than the glass transition temperature (Tg = −27.3 °C) of the PVC-P GMB, placing the material in the glassy state regime. At low tensile rates, the extended loading duration allows molecular chains limited time for local relaxation, which delays microcrack propagation and yields a greater fracture strain. In contrast, under high tensile rates, the extremely short loading duration prevents chain segment relaxation, inducing premature brittle fracture. Consequently, both fracture strength and fracture strain decrease simultaneously with increasing loading rate. Combining the results of the two-factor analysis in Figure 8 reveals that the temperature–tensile rate interaction at −40 °C is substantially stronger than that at ambient temperatures, indicating that the ultra-low temperature environment significantly amplifies the sensitivity of mechanical properties to loading rate. Furthermore, the slope of the stress–strain curve increases markedly with decreasing temperature, indicating that the stiffness (elastic modulus) is significantly enhanced, and its resistance to elastic deformation is improved (while its deformability is correspondingly reduced). This behavior is attributed to the suppression of polymer segmental motion at low temperatures: as temperature decreases, the thermal motion of molecular chains weakens and segmental relaxation times are prolonged. During tensile loading, energy dissipation via chain slippage or orientation becomes difficult, which in turn causes the material to exhibit greater rigidity and strength.

The fracture strain decreases significantly with decreasing temperature. At 20 °C, the GMB exhibits high toughness, with fracture strains exceeding 140% at all tensile rates with the exception of 1 mm/min, demonstrating good plastic deformation. However, as the temperature decreases, the fracture strain shows a continuous downward trend. Notably, at a tensile rate of 100 mm/min (Figure 8e), the fracture strain decreased from 145.11% to 78.57% as the temperature dropped from 20 °C to −40 °C, representing a reduction of 45.85%. This indicates that the PVC-P GMBs transition from a highly ductile to a brittle state. The low-temperature substantially weakens its plastic deformation capacity, leading to reduced ductility and enhanced brittleness.

Table 4 and Table 5 quantitatively present the effects of test temperature on the mechanical properties of the PVC-P GMBs. The results demonstrate that test temperature exerts a significant effect on both fracture strength and fracture strain. Fracture strength first increases and then decreases with rising temperature at moderate and high tensile rates, and increases with rising temperature at 1 mm/min. whereas fracture strain exhibits a clear negative correlation with temperature. Taking a tensile rate of 1 mm/min as an example, compared with the reference condition at 20 °C, the fracture strength reach its maximum when the temperature drops to −40 °C. The fracture strength increases continuously as the temperature decreases from 20 °C to −30 °C, with increase rate ranging from −6.62% to −38.00%. When the tensile rate increased to 100 mm/min, a temperature decrease from 20 °C to −30 °C results in an increase in fracture strength of up to 26.74%. The fracture strength at −40 °C is markedly lower than those measured at other temperatures. Furthermore, as the tensile rate increases, the strength-enhancing effect of low temperature is further amplified. This phenomenon is attributed to the viscoelastic properties of the PVC-P GMB: as temperature decreases, the thermal motion of polymer molecular segments is significantly suppressed and segmental relaxation times are substantially prolonged, making energy dissipation through slippage or orientation during tensile loading difficult, thereby markedly increasing the material’s stiffness and deformation resistance—manifested macroscopically as enhanced fracture strength. Concurrently, an interaction effect exists between tensile rate and temperature: at high tensile rates, segmental relaxation is further restricted, and the material tends to exhibit more pronounced glassy mechanical behavior, thereby reinforcing the strengthening effect of low-temperature conditions.

As shown in Table 5, the fracture strain generally decreases monotonically with decreasing temperature, indicating that temperature exerts a significant effect on the material’s plastic deformation capacity. Taking a tensile rate of 1 mm/min as an example, compared with the reference condition at 20 °C, the fracture strain decreases by 44.16% when the temperature drops to −40 °C; when the tensile rate is increased to 100 mm/min, a temperature decline from 20 °C to −40 °C results in a decrease of 66.98% in fracture strain. This indicates that the detrimental effect of low temperature on material ductility intensifies with increasing tensile rate. The underlying mechanism is as follows: decreasing temperature inhibits the mobility of polymer molecular segments, impairing their capacity for slippage and orientation, which leads to a significant reduction in plastic deformability. When the temperature is further reduced to near or below the glass transition temperature, the material transitions from a highly elastic to a glassy state, exhibiting increased brittleness and a substantial decrease in fracture strain. At the same time, high tensile rates further shorten the response time of molecular segments, exacerbating the embrittlement tendency at low temperatures and making the reduction in fracture strain even more pronounced.

**Figure 9:**
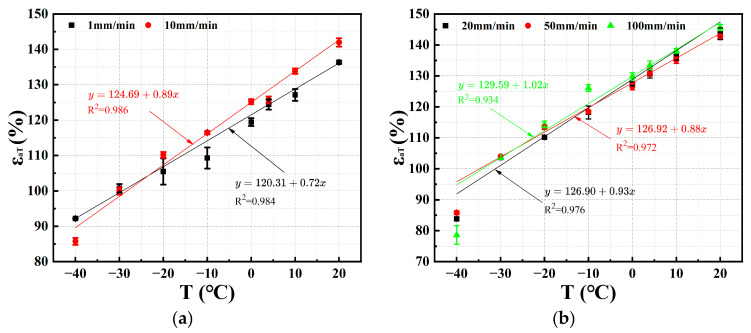
Linear fit of fracture strain versus temperature: (**a**) 1, 10 mm/min; (**b**) 20, 50, 100 mm/min.

Figure 9 presents the fitted curves of fracture strain against temperature for the PVC-P GMBs, Table 6 presents the intercepts, slopes, residual sum of squares (RSS), Pearson’s correlation coefficients, and R-squared (COD) values for the linear fits of each group. Under all tensile rates, the fracture strain exhibits a linear relationship with the test temperature, with the coefficients of determination (R^2^) exceeding 0.934 across all tensile rates and most surpassing 0.97. This indicates a strong positive linear correlation, demonstrating that temperature primarily accounts for the variation in fracture strain and that the linear model effectively characterizes the material’s plastic response under the tested conditions. In terms of the fitting parameters, both the intercept and the slope generally increase with increasing tensile rate. A steeper slope corresponds to a faster increase in fracture strain with temperature. Notably, at a tensile rate of 100 mm/min, the rate of increase in fracture strain is significantly greater than that at the other rates.

**Figure 10:**
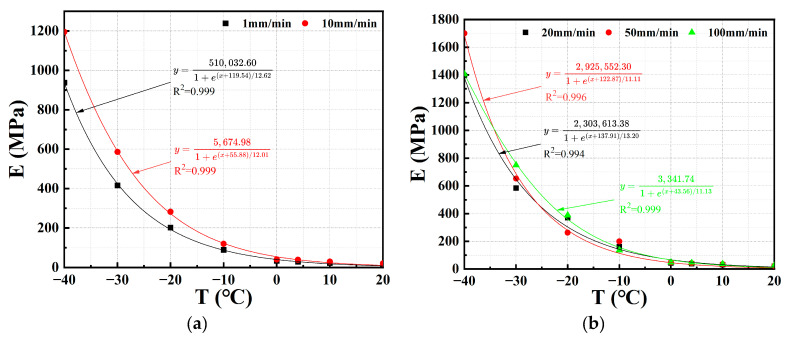
Boltzmann fit of elastic modulus versus temperature: (**a**) 1, 10 mm/min; (**b**) 20, 50, 100 mm/min.

Figure 10 presents the Boltzmann-fitted curves of the elastic modulus of the PVC-P GMBs as a function of temperature, while Table 7 shows the Boltzmann model fitting parameters, chi-squared value, and COD values corresponding to each stretching rate. Under all tensile rates, the elastic modulus exhibits a S-shaped nonlinear relationship with temperature. It remains relatively high in the low-temperature range, decreases sharply with increasing temperature, and then stabilizes in the high-temperature range, demonstrating pronounced sensitivity to temperature. To quantify this nonlinear relationship, the Boltzmann function was employed to fit the experimental data at different tensile rates, expressed as follows:

(6) $$y=\frac{A_{1}-A_{2}}{1+e^{(x-x_{0})/dx}}+A_{2}$$

In this model, A_1_ corresponds to the asymptotic value of the elastic modulus on the low-temperature plateau, A_2_ corresponds to the asymptotic value on the high-temperature plateau, X_0_ is the midpoint temperature of the transition region, and dx governs the steepness of the transition.

**Table 7:** Boltzmann fitting parameters for the elastic modulus as a function of temperature.

Tensile Rate (mm/min)	Equation	A1	A2	X_0_	dx	Reduced Chi-Sqr	R-Squared (COD)
1	$y=\frac{A_{1}-A_{2}}{1+e^{(x-x_{0})/dx}}+A_{2}$	51,0032.60 ± 2.72 × 10^7^	0	−119.55 ± 677.57	12.63 ± 0.67	54.332	0.999
10	$y=\frac{A_{1}-A_{2}}{1+e^{(x-x_{0})/dx}}+A_{2}$	5674.99 ± 1939.27	0	−55.88 ± 5.89	12.02 ± 0.56	85.744	0.999
20	$y=\frac{A_{1}-A_{2}}{1+e^{(x-x_{0})/dx}}+A_{2}$	2,303,613.38 ± 1.49 × 10^9^	0	−137.91 ± 8548.88	13.20 ± 2.78	1990.635	0.994
50	$y=\frac{A_{1}-A_{2}}{1+e^{(x-x_{0})/dx}}+A_{2}$	2925,552.30 ± 1.84 × 10^9^	0	−122.87 ± 7008.22	11.11± 2.19	1899.172	0.996
100	$y=\frac{A_{1}-A_{2}}{1+e^{(x-x_{0})/dx}}+A_{2}$	3341.75 ± 751.15	0	−43.56 ± 4.50	11.13 ± 0.86	408.797	0.999

The coefficients of determination (R^2^) exceed 0.994 at all tensile rates, indicating that the Boltzmann model characterizes the nonlinear temperature-dependent variation of elastic modulus with a high accuracy. In terms of the fitting parameters, X_0_ shifts toward higher temperatures with increasing tensile rate; dx remains between 11.11 and 13.20 across all rates, indicating that the steepness of the transition is insensitive to tensile rate, and the elastic modulus exhibits a consistent S-shaped pattern at different rates.

#### 3.2.4. Coupling Interaction of Temperature–Tensile Rate Interaction

**Figure 11:**
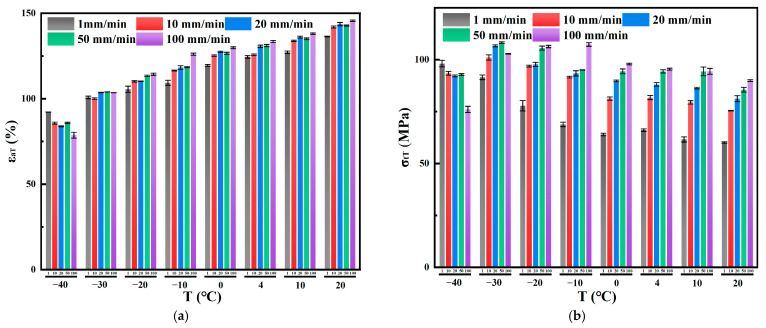
Two-factor analysis: (**a**) true fracture strain; (**b**) true fracture stress.

Figure 11 illustrates the variations in true fracture strain and true fracture stress of the PVC-P GMB under coupled temperature–tensile rate conditions, visually demonstrating the synergistic effects of these two factors.

Regarding the temperature effect, across all five tensile rates, the true fracture strain exhibits a consistent increasing trend with rising temperature. In contrast, the true fracture stress decreases with increasing temperature at the low tensile rate of 1 mm/min, while showing an initial increase followed by a decrease at medium-to-high rates. Low-temperature conditions significantly enhance fracture strength while simultaneously suppressing plastic deformability, indicating pronounced low-temperature embrittlement.

The rate-dependent behavior exhibits a temperature threshold effect. In the temperature range of −10 °C to 20 °C, both true fracture strain and fracture stress generally increase with rising tensile rate. However, at the extreme low temperature of −40 °C, this trend reverses-excessively high tensile rates lead to a reduction in fracture parameters, reflecting a brittle fracture mechanism induced by molecular chain freezing under loading at ultra-low temperatures.

Temperature and tensile rate do not act independently; rather, they exhibit a significant coupled interaction. The temperature level modulates the material’s sensitivity to loading rate: within the ambient temperature range, the rate-induced variation in mechanical properties is relatively moderate; upon entering the low-temperature regime, the rate-induced performance differences become increasingly pronounced, with rate sensitivity peaking at −40 °C. These findings indicate that experimental results obtained under a single temperature or a single tensile rate are insufficient to fully characterize the mechanical behavior of PVC-P GMBs; the coupled effects of both temperature and tensile rate must be considered simultaneously.

## 4. Conclusions

This study investigated the effects of test temperature (−40, −30, −20, −10, 0, 4, 10, and 20 °C) and tensile rate (1, 10, 20, 50, and 100 mm/min) on the axial tensile properties of PVC-P GMBs. The following main conclusions were drawn:

1. Decreasing temperature increases the tensile strength and elastic modulus of the PVC-P GMBs while reducing its strain at break, resulting in a high-strength but brittle behavior. Conversely, increasing temperature enhances chain segment mobility, leading to reduced strength and modulus but improved deformability, manifesting as low-strength but ductile behavior. Temperature sensitivity is coupled with loading rate effects, and together they govern the mechanical response. In cold-region hydraulic engineering, particular attention should be paid to the risks of toughness degradation and brittle fracture induced by low temperatures.
2. In cold-region applications, the actual service temperature of PVC-P GMBs is typically lower than laboratory test temperatures, resulting in mechanical properties that differ significantly from those obtained at room temperature. Design based solely on room-temperature parameters may underestimate the risk of low-temperature embrittlement. Therefore, specialized experimental investigations should be conducted at the anticipated service temperatures to elucidate the evolution of low-temperature performance and to provide a basis for engineering safety.
3. The mechanical properties of PVC-P GMBs are dependent on tensile rate. In the low-rate range (1–20 mm/min), fracture strength, fracture strain, and elastic modulus increase significantly with increasing tensile rate; in the medium-to-high-rate range (20–100 mm/min), the rate effect tends to saturate. Extremely low tensile rates are prone to inducing creep interference, whereas relatively high rates may compromise test stability. Tensile rates of 10 mm/min or 20 mm/min are therefore recommended for future testing.
4. Design in high-altitude cold regions should be based on the lowest anticipated service temperature and the most unfavorable loading rate, with due consideration of the coupled effects of temperature and tensile rate. It is recommended that a low-temperature mechanical property evaluation system be established, or that a constitutive model incorporating both temperature and rate variables be developed, to enable prediction of critical mechanical parameters.

## Figures and Tables

**Table 1:** Physical and mechanical properties of tested PVC-P GMBs.

Property	Method	Value	Unit
Thickness	ASTM D5199, 2025 [17]	1.5 ± 0.2	mm
Mass per unit area	ASTM D5261-10, 2024 [24]	0.22	g/cm^2^
Break strength	ASTM D6693/D6693M-25, 2025 [25]	157.52	MPa
Yield strength	ASTM D6693/D6693M-25, 2025 [25]	12.07	MPa
Break elongation	ASTM D6693/D6693M-25, 2025 [25]	133.54	%
Yield elongation	ASTM D6693/D6693M-25, 2025 [25]	53.02	%

**Table 2:** Nominal fracture strain of PVC-P GMBs at different tensile rates (unit: %).

Test Temperature (°C)	Tensile Rate (mm/min)								
	50	1	10	20	100	ε_1−50_	ε_10−50_	ε_20−50_	ε_100−50_
20	317.65	289.36	316.79	325.17	326.79	−28.29	−0.86	7.52	9.14
10	283.21	256.12	284.13	293.27	299.51	−27.09	0.92	10.06	16.30
4	271.33	248.64	252.00	265.73	282.93	−22.69	−19.33	−5.60	11.60
0	256.60	232.76	251.54	258.55	268.39	−23.84	−5.06	1.95	11.79
−10	228.07	195.18	218.87	229.48	255.64	−32.89	−9.20	1.41	27.57
−20	210.48	184.92	201.95	199.93	215.33	−25.56	−8.53	−10.55	4.85
−30	183.06	170.37	174.45	182.56	181.41	−12.69	−8.61	−0.50	−1.65
−40	136.41	151.31	135.21	131.62	125.14	14.90	−1.20	−4.79	−11.27

**Table 3:** Nominal fracture strength of PVC-P GMBs at different tensile rates (unit: MPa).

Test Temperature (°C)	Tensile Rate (mm/min)								
	50	1	10	20	100	σ_1−50_	σ_10−50_	σ_20−50_	σ_100−50_
20	20.52	15.34	18.39	19.69	21.07	−5.18	−2.13	−0.83	0.55
10	22.29	16.75	21.05	22.02	23.77	−5.54	−1.24	−0.27	1.48
4	25.16	18.73	23.79	24.02	25.77	−6.43	−1.37	−1.14	0.61
0	26.77	19.33	23.32	25.17	26.86	−7.44	−3.45	−1.60	0.09
−10	29.08	23.73	28.30	28.53	30.51	−5.35	−0.78	−0.55	1.43
−20	34.02	27.00	32.12	32.22	33.66	−7.02	−1.90	−1.80	−0.36
−30	38.18	33.04	37.04	37.51	36.38	−5.14	−1.14	−0.67	−1.80
−40	39.27	37.06	39.14	39.71	34.22	−2.21	−0.13	0.44	−5.05

**Table 4:** Fracture strength of PVC-P GMBs at different tensile rates (Unit: MPa).

Test Temperature (°C)	Tensile Rate (mm/min)				
	1	10	20	50	100
20	60.10	75.44	81.21	85.51	89.91
10	61.57	79.43	86.31	94.35	94.38
4	66.05	81.73	88.06	94.35	95.43
0	63.94	81.26	89.75	94.37	97.92
−10	68.81	91.61	93.39	95.07	107.30
−20	77.79	96.90	97.72	105.57	106.33
−30	91.48	101.05	106.63	108.18	102.77
−40	98.10	93.42	92.16	92.80	76.03

**Table 5:** Fracture strain of PVC-P GMBs at different tensile rates (unit: %).

Test Temperature (°C)	Tensile Rate (mm/min)				
	1	10	20	50	100
20	136.36	141.95	143.63	142.76	145.62
10	127.11	133.86	136.05	135.06	138.08
4	124.44	125.68	130.67	131.10	133.43
0	119.45	125.20	127.41	126.55	129.83
−10	109.30	116.47	118.20	118.44	126.05
−20	105.47	110.17	110.17	113.39	114.32
−30	100.74	100.00	103.62	103.99	103.56
−40	92.20	85.75	83.86	85.82	78.64

**Table 6:** Linear fitting parameters for fracture strain versus temperature.

Tensile Rate (mm/min)	Equation	Intercept	Slope	RSS	Pearson’s r	R-Squared (COD)
1	y = a + b × x	120.31 ± 0.78	0.72 ± 0.04	24.481	0.992	0.984
10	y = a + b × x	124.69 ± 0.91	0.89 ± 0.04	33.582	0.993	0.986
20	y = a + b × x	126.90 ± 1.25	0.93 ± 0.06	63.132	0.988	0.976
50	y = a + b × x	126.92 ± 1.29	0.88 ± 0.06	67.583	0.986	0.972
100	y = a + b × x	129.59 ± 2.31	1.02 ± 0.11	216.61	0.967	0.934

## Data Availability

The original contributions presented in this study are included in the article. Further inquiries can be directed to the corresponding author.

## Abbreviations

The following abbreviations are used in this manuscript:

PVC-P	Plasticized polyvinyl chloride
GMB	Geomembrane
DIC	Digital image correlation
HDPE	High-density polyethylene
DBTT	Ductile-to-brittle transition temperature
Tg	Glass transition temperature
DMA	Dynamic Mechanical Analysis
PID	Proportional-Integral-Derivative
RSS	Sum of squares
COD	R-squared
